# Structural Sampling of Glycan Interaction Profiles Reveals Mucosal Receptors for Fimbrial Adhesins of Enterotoxigenic *Escherichia coli*

**DOI:** 10.3390/biology2030894

**Published:** 2013-07-01

**Authors:** Emanuela Lonardi, Kristof Moonens, Lieven Buts, Arjen R. de Boer, Johan D. M. Olsson, Manfred S. Weiss, Emeline Fabre, Yann Guérardel, André M. Deelder, Stefan Oscarson, Manfred Wuhrer, Julie Bouckaert

**Affiliations:** 1 Center for Proteomics and Metabolomics, Leiden University Medical Center, P.O. Box 9600, RC Leiden 2300, The Netherlands; E-Mails: e.lonardi@lumc.nl (E.L.); ardeboer@gmail.com (A.R.B.); a.m.deelder@lumc.nl (A.M.D.); m.wuhrer@lumc.nl (M.W.); 2 Structural & Molecular Microbiology, VIB Department of Structural Biology, Brussels 1050, Belgium; E-Mail: kristof.moonens@vub.ac.be; 3 Molecular Recognition, VIB, Brussels 1050, Belgium; E-Mail: lievbuts@vub.ac.be; 4 Vrije Universiteit Brussel, Pleinlaan 2, Brussels 1050, Belgium; 5 Centre for Synthesis and Chemical Biology, University College Dublin, Belfield, Dublin 4, Ireland; E-Mails: johan.olsson@biochromix.com (J.D.M.O.); stefan.oscarson@ucd.ie (S.O.); 6 Helmholtz-Zentrum Berlin für Materialien und Energie, Institute for Soft Matter and Functional Materials, Macromolecular Crystallography (HZB-MX), Albert-Einstein-Strasse 15, Berlin D-12489, Germany; E-Mail: manfred.weiss@helmholtz-berlin.de; 7 Unité de Glycobiologie Structurale et Fonctionnelle (UGSF), Université Lille 1, UMR8576 du CNRS, Villeneuve d’Ascq 59655, France; E-Mails: emeline.fabre@univ-lille1.fr (E.F.); yann.guerardel@univ-lille1.fr (Y.G.)

**Keywords:** adhesin, glycan array, *E. coli*, enterotoxigenic, F17G, FedF, FimH, charge, arginine, sulfate

## Abstract

Fimbriae are long, proteinaceous adhesion organelles expressed on the bacterial envelope, evolutionarily adapted by *Escherichia coli* strains for the colonization of epithelial linings. Using glycan arrays of the Consortium for Functional Glycomics (CFG), the lectin domains were screened of the fimbrial adhesins F17G and FedF from enterotoxigenic *E. coli* (ETEC) and of the FimH adhesin from uropathogenic *E. coli*. This has led to the discovery of a more specific receptor for F17G, GlcNAcβ1,3Gal. No significant differences emerged from the glycan binding profiles of the F17G lectin domains from five different *E. coli* strains. However, strain-dependent amino acid variations, predominantly towards the positively charged arginine, were indicated by sulfate binding in FedF and F17G crystal structures. For FedF, no significant binders could be observed on the CFG glycan array. Hence, a shotgun array was generated from microvilli scrapings of the distal jejunum of a 3-week old piglet about to be weaned. On this array, the blood group A type 1 hexasaccharide emerged as a receptor for the FedF lectin domain and remarkably also for F18-fimbriated *E. coli*. F17G was found to selectively recognize glycan species with a terminal GlcNAc, typifying intestinal mucins. In conclusion, F17G and FedF recognize long glycan sequences that could only be identified using the shotgun approach. Interestingly, ETEC strains display a large capacity to adapt their fimbrial adhesins to ecological niches via charge-driven interactions, congruent with binding to thick mucosal surfaces displaying an acidic gradient along the intestinal tract.

## 1. Introduction

Bacterial adhesion to mammalian tissue constitutes the first step in host colonization, through specific structures known as fimbriae and pili. F17 and F18 fimbriae are wire-like structures expressed on the surface of enterotoxigenic and septicemic *Escherichia coli* (ETEC) strains that secrete toxins causing diarrhea in infantile livestock [1]. The long and flexible F17 and F18 have an overall morphology and carbohydrate specificity clearly distinguishable from the rigid, rod-shaped type-1 and P pili from uropathogenic *E. coli* strains, and easily reach in between the digiform brush border microvilli of intestinal epithelia [2,3]. F17, F18 and type-1 fimbriae are assembled by the chaperone/usher biogenesis pathway [4] and comprise a few thousand copies of a major pilin (F17A [5], FedA and FimA respectively), several minor pilin proteins and a single two-domain adhesin (TDA) at the tip (F17G [6], FedF [7] and FimH, respectively). The *N*-terminal domain of TDAs is a lectin domain, also called receptor-binding domain, because it mediates adherence of fimbriated bacteria to glycoconjugate receptors on host epithelial linings [8]. 

Indeed, the key information for the unique interaction between the fimbrial adhesin and the host cell lies in the recognition of distinct glycan receptors on the host cell itself and is highly specific [9,10]. For example, FimH is the TDA of type-1 pili and interacts with glycoproteins on the bladder wall [8]. The optimal receptor epitope, recognized by FimH with the highest affinity, is the exposed Manα1, 3Manβ1, 4GlcNAcβ1, 4GlcNAc structure of *N*-linked high-mannose type glycans [11]. F17 and F18 fimbriated ETEC infect respectively neonatal ruminants (goat kids, lambs and calves, dependent on the particular *E. coli* strain) and just-weaned piglets. Although, F17-fimbriae mediated bacterial adhesion can be inhibited by the monosaccharide *N*-acetylglucosamine (GlcNAc) [12], each F17-related adhesin probably recognizes a different receptor on host tissues, as reflected in the different isolation sources of the strains [13]. For example, the ETEC strain expressing F17b fimbriae was isolated from septicaemic calves and lambs [14], whereas F17a fimbriae are produced on bovine ETEC strains [5]. Clinical strains of F17G display amino acid sequence variation of up to 10% and six natural variants (termed a–f) of the F17 operon have been previously identified [6,14,15]. For each of these, except for variant d, the receptor-binding domain has been crystallized in its apo-form or in complex with a carbohydrate ligand [16,17,18]. However, still today, the role of variation in the amino acid sequences of the lectin domains within a single fimbrial system remains poorly understood. 

The specificity profiles of the lectin domains of five of the F17G variants, one FedF variant (strain 107/86 [19]) and FimH were analyzed on two different array platforms: synthetic [20] and natural [21] glycan arrays, and a shotgun glycan array has been tailored for screening with the FedF lectin. The synthetic glycan arrays are represented by printed arrays versions 2 and 4.1 from the Consortium for Functional Glycomics (CFG). The natural glycan array is an array displaying a diverse set of partially purified, partially characterized glycans from various sources, yet not necessarily biologically relevant ones [21,22,23]. The shotgun array has been constructed with glycans isolated from FedF adhesin-receptive target tissue as the starting material. 

The natural receptor for the F17G adhesin could, as such, be more specificallly determined, beyond the monosaccharide β-d-GlcNAc. The oligosaccharides recognized by F17G share a terminal non-reducing GlcNAcβ1,3Gal disaccharide. A crystal structure of the F17G lectin domain, variant b, in complex with GlcNAcβ1,3Gal, has been refined to high-resolution. Surface Plasmon Resonance (SPR) interaction studies using a newly synthesized GlcNAcβ1-xGal series (x being 2, 3, 4 or 6) and F17G crystal structures in complex with these disaccharides provide insight in the structural basis of the variant-specific binding profiles. Amino acid sequence and crystal structure analyses of the lectin domains of F17G, FedF and FimH elucidate a previously unacknowledged basis for strain-dependent glycan binding profiles of *E. coli* fimbrial adhesins, one that is driven by electrostatic interactions.

## 2. Experimental Procedures

### 2.1. Expression and Purification of Two-Domain Adhesin Lectin Domains

F17aG, F17bG, F17dG, F17eG lectin domains were purified from *E. coli* periplasmic extracts as described previously [16,18]. Briefly, affinity chromatography on GlcNAc—6% beaded agarose (Sigma) and elution with 200 mM GlcNAc was followed by gel filtration (Superdex 75 HR) in 20 mM Tris pH 8 and 150 mM NaCl to clear the protein from GlcNAc. The FedF and FimH adhesins were purified using ion exchange chromatography [24,25,26].

### 2.2. CFG Glycan Microarray Screening

A microarray presenting 264 different glycans (mammalian printed array version 2) was used to evaluate if the F17G variant lectin domains differ in the range of glycan receptors that they recognize, thereby establishing tropism. The microarray was set up within the Consortium for Functional Glycomics (CFG) ([20], and supplemental data). Binding of F17G (200 µg/mL) was detected using anti-F17G polyclonal rabbit IgG (10 µg/mL), followed by fluorescently labelled goat anti-rabbit polyclonal antibodies (IgG-488, 5 µg/mL). 

With mammalian printed array version 4.1, a new evaluation parameter was taken into account, namely % CV = 100 × Std. Dev/Mean. The highest and the lowest point from each set of six replicates have been removed so the average is of 4 values rather than 6. This eliminates some of the false hits that contain a single very high or low point. Thus, points with a high % CV should be considered suspect. Another update with version 4.1 arrays was separate screening results for the primary anti-lectin antibodies that were used for detection, to enable subtraction of positive data from the lectin responses. All data have been analyzed with regard to what glycan structures the sample binds, as well as what related structures it does not bind. This comparison provides information on binding specificity. The CFG glycan array data are available containing searchable results by means of flex-based data browser tools [27].

### 2.3. Synthesis of a GlcNAcβ1-xGal Disaccharide Series

The four β-d-GlcNAc-(1→2, 3, 4 or 6)-β-d-Gal-(1-O)Me disaccharides have been synthesized using the same glycosyl donor, *i.e.*, ethyl 3,4,6-tri-*O*-benzyl-2-deoxy-2-phthalimido-1-thio-β-d-glucopyranoside (ESI Scheme S1) [28] (**1**). NIS/AgOTf-promoted couplings between donor **1** and the known galactose acceptors **2** [29] (2-OH), **3** [30] (3-OH), **4** [31] (4-OH) and **5** [32] (6-OH) afforded the desired β-linked disaccharides **6** (74%), **7** (70%), **8** (54%) and **9** (51%), respectively. Transformation of the phtalimido group in these derivatives into an acetamido group has been accomplished by treatment with diethylamine in EtOH followed by acetylation of the obtained intermediate amino group with acetic anhydride. Deprotection by catalytic hydrogenolysis then afforded the target structures **10** (39%), **11** [33] (40%), **12** (41%) and **13** [34] (37%). All other carbohydrates have been purchased from Dextra Laboratories, UK.

### 2.4. Surface Plasmon Resonance Measurements for Linkage Discrimination by F17G

The affinity profiles were determined by surface plasmon resonance (SPR) measurements using a *BiaCore 3000* instrument (GE Healthcare). This method has been proven sufficiently sensitive to detect even the binding of a single monosaccharide (MW = 180 Da) to immobilized 17-kDa F17G protein [18]. In order to ensure adequate and consistent immobilization levels, variants of the lectin domains with oligolysine (4Lys) tails have been constructed. The proteins were covalently immobilized on CM5 sensor chips (Biacore AB) to an average density of 2000 RU (resonance units in pg/mm^2^). On each chip, one flow cell was coated with a camel single domain antibody for use as a reference cell. The other three flow cells (Fc2, Fc3 and Fc4) were coated with three different F17G variants. Carbohydrate concentrations ranging from 10 µM to 10 mM in running buffer (20 mM Hepes, pH 7.4; 150 mM NaCl; 3 mM EDTA and 0.005% surfactant Tween 20) were incubated for one minute on all flow cells simultaneously, at 25 °C and at a flow rate of 30 µL/min. Complete dissociation of the carbohydrates in running buffer was verified before starting a new binding cycle. All binding cycles were performed in duplicate, including a zero concentration cycle (injection of pure running buffer). All analysis was done using the BIAeval software. In each case the equilibrium resonance signal from the reference cell was subtracted from that of the various F17G flow cells (2-1, 3-1, 4-1). A Langmuir binding isotherm with a 1:1 stoichiometry was fitted to the data, from which the equilibrium dissociation constants, K*_d_*, were obtained.

### 2.5. Shotgun Glycan Microarray Production

To investigate the exact carbohydrate nature of the putative F18 fimbrial receptor, a shotgun glycan array was produced at the Leiden University Medical Center. Scrapings of the distal part of the jejunum were obtained from a three-week old male piglet (Pietrain stress-negative), as in this age period piglets are most susceptible to ETEC and STEC infections causing post-weaning diarrhea or edema disease. In short, glycoproteins and glycolipids were isolated from the starting material by sonication and solvent extraction. Porcine intestinal *N*-glycans and glycolipid-derived glycan moieties (l-glycans) were prepared by enzymatically treating both fractions with glycosidases (respectively peptide *N*-glycanase F and endoglycosylceramidase II) as described previously [23]. In a subsequent clean-up step, l-glycans were separated into acidic and neutral type glycans [23]. Glycan pools were labeled with the fluorescent dye 2-aminobenzoic acid (AA) by reductive amination [21], followed by hydrophilic liquid interaction chromatography (HILIC) fractionation (neutral lipid glycan: 87 fractions; acidic lipid glycans: 87 fractions and *N*-glycans: 102 fractions). The attached fluorescent AA-tag allowed the detection and determination of the concentration of glycans in each fraction, as well as directional immobilization (via their secondary aromatic amine generated during reductive amination) onto epoxy-coated slides. The fluorescently labeled glycans were printed in triplicate and printed slides were stored overnight at room temperature in a humidity chamber to prevent drying of the spots while immobilization chemistry was completed. Finally, slides were left to dry completely and stored in sealed boxes at room temperature, protected from light, until used [23]. 

### 2.6. Natural and Shotgun Glycan Microarrays Screening

All five F17G variant receptor binding domains (a, b, d, e, f), FedF, and FimH, were screened on pre-designed glycan arrays displaying natural *N*-linked glycans as well as on the shotgun microarrays designed from F18-fimbriated *E. coli* target tissues. Briefly, between the several printed arrays present on a glass slide, thin-film-like hydrophobic barriers were drawn with a PAP-PEN (RPI, Mount Prospect, IL, USA). The barriers created a proper surface tension to hold solutions within the array area. Compounds that did not bind to the glass slide during printing were washed away by extensive rinsing with 0.05% Tween20 in PBS. Remaining active epoxide groups were blocked by 4% BSA, 0.05% Tween20 in PBS for 90 min at room temperature. Subsequently, the slides were rinsed with PBS. Each microarray was incubated with the protein of interest in 0.01% Tween20 in PBS with 1% BSA for 90 min at room temperature. Slides were then washed with successive rinses in 0.05% Tween20 in PBS and PBS. Detection of bound lectin domains was achieved with a typical sandwich assay, in which the primary antibody was always a rabbit polyclonal IgG (10 µg/mL, in PBS with 0.01% Tween20 and 1% BSA), incubated for 45 min at room temperature, followed by incubation with mouse anti-rabbit AlexaFluo555 labeled antibodies (at 1:1,000 dilution, Molecular Probes). During incubation, the slides were protected from light and an extensive wash step with 0.05% Tween20 in PBS and 0.01% Tween20 in PBS was performed between each incubation step. Finally, slides were rinsed with 0.05% Tween20 in PBS, PBS, and H_2_O and dried under nitrogen. In a second approach, 6-histidine (His)-tagged F17G constructs for variants a and b were used to reduce background noise and mouse anti-His-tag antibodies were used for detection.

Fluorescence signals were integrated and plotted against fraction numbers. Data were retroactively corrected for false positives. An antibody control was performed for each individual experiment without prior binding of the protein of interest and observed positive fractions were removed from the final data. Data points were omitted in cases where the standard deviation surpassed half of the value of the average fluorescence signal. In the case of shotgun glycan microarray incubations, those HILIC fractions responding positively were structurally characterized using tandem mass spectrometry [23], and glycan structures were verified by comparison of fragmentation patterns with those of commercial standards [35]. The shotgun array was also screened with the lectin domain of FimH as a positive quality control on the glycan structures present in the HILIC fractions [36].

### 2.7. Crystallization and Structure Solution

F17bG was crystallized at a concentration of 12 mg/mL with 10 mM GlcNAcβ1,3Gal using hanging drop vapour diffusion against 1 M Li_2_SO_4_, 0.1 M Tris-HCl at pH 8.5 and 10 mM NiCl_2_, as described in [16]. Analysis of the corresponding diffraction data using the Phenix XTriage module indicated the presence of merohedral twinning. Consequently, all refinement steps were carried out using the amplitude-based twinning correction target (TWIN_LSQ_F) in the Phenix refinement program, with a twin law of h,-h-k,-l and a final refined twinning fraction of 0.41. The ligand-free FedF structure is a further refinement of PDB entry 4B4P [25], from a bromide-soaked FedF crystal originally used for MAD phasing [37].

## 3. Results

### 3.1. Synthetic and Natural Glycan Microarray Screening

**Figure 1:**
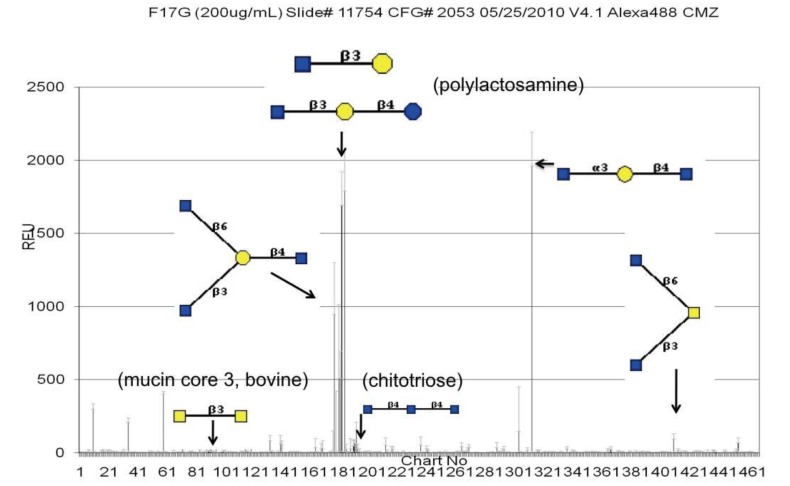
CFG synthetic glycan array showing the F17aG specificity profile (all data are available in a browsable format [27].

A microarray presenting 264 different glycans (mammalian printed array version 2 from the CFG, experiment 400) was used to evaluate whether the F17G variant lectin domains differ in the range of glycan receptors that they can recognize, thereby establishing strain-dependent tropism. Table 1 displays the results in Relative Fluorescence Units (RFU) measurements for six glycan replicates per single experiment. The glycan array screening has been repeated for F17aG only on printed array version 4.1 (experiment 2053) (Figure 1).

The results clearly showed that GlcNAcβ1,3Galβ1-terminating glycans consistently bind well to each of the variant F17G lectin domains (deposited at 200 μg/mL, Table 1; Figure 1). Certain glycan structures did not give reproducible RFU signals and were not always recognized on both versions of the array, including a mucin core 3 (glycan 88 in Table 1, that lacks signal, or GalNAcβ1,3GalNAc in Figure 1).

**Figure 2:**
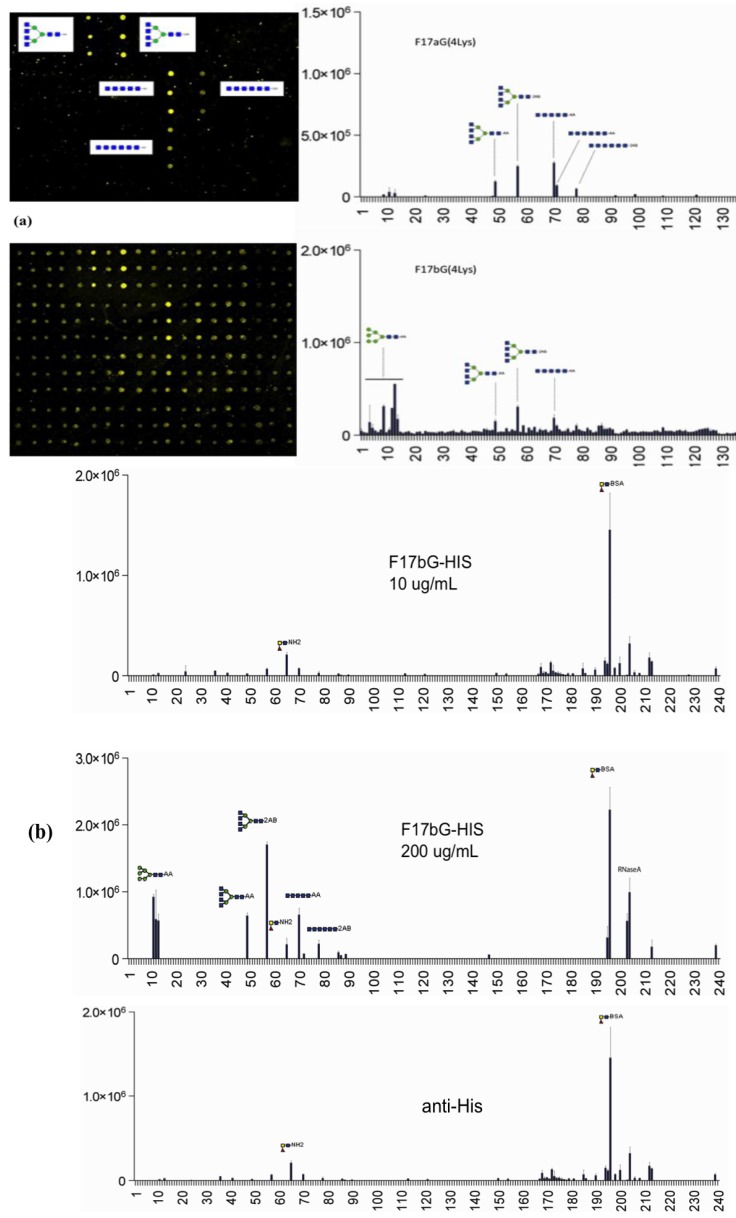
(**a**) Natural glycan array screening for F17G(4Lys) variants a (upper) and b (lower); (**b**) Natural glycan array concentration-dependent screening for F17bG with His-tag. Mouse anti-His antibodies, instead of polyclonal rabbit anti-F17G, were here used as primary antibodies, in order to reduce the background level observed in (**a**) for F17bG.

Screening results for all five F17G variant lectin domains (a, b, d, e, f) on the natural glycan arrays [21], either using F17G constructs containing four *C*-terminal lysine residues (Figure 2a) or six *C*-terminal histidines (Figure 2b), were congruent with the results from the CFG array (Figure 1). From our natural glycan array screening, it appears that higher applied F17G concentrations are important for good signals (Figure 2). This is in contrast with the control adhesin FimH that consistently gives high signals for oligomannoside structures at 10 μg/mL concentrations. The high concentration needed of F17G correlates with its low affinity for the GlcNAcβ1,3Gal epitope.

**Table 1:** Binding of five F17G variants on the Consortium for Functional Glycomics (CFG) mammalian printed glycan array. Values are Relative Fluorescence Units (RFU), averaged over six replicates, for the best glycan binders to five F17G variant sequences on the CFG glycan array version 2. Standard errors of the means (SEM) are presented in parentheses.

N°	Glycan name	F17aG	F17bG	F17dG	F17eG	F17fG
167	GlcNAcβ1,3Galβ1,4Glcβ-Sp0	57,013 (5,191)	13,149 (2,286)	29,181 (3,281)	54,005 (5,715)	26,336 (7,255)
165	GlcNAcβ1-3Galβ1,4GlcNAcβ-Sp8	51,880 (5,023)	15,483 (1,729)	17,991 (4,401)	49,223 (5,377)	28,888 (6,925)
160	GlcNAcβ1,3(GlcNAcβ1,6)Galβ1,4GlcNAcβ-Sp8	44,391 (3,949)	21,630 (2,490)	17,195 (3,442)	41,907 (4,694)	37,083 (5,973)
159	GlcNAcβ1,3(GlcNAcβ1,6)GalNAcα-Sp8	35,858 (4,079)	12,183 (1,524)	23,756 (2,105)	46,840 (6,520)	37,083 (1,197)
162	GlcNAcβ1,3Galβ-Sp8	32,288 (4,175)	16,310 (1,907)	16,277 (9,959)	38,836 (5,631)	27,648 (1,137)
161	GlcNAcβ1,3GalNAcα-Sp8	25,971 (3,174)	15,182 (2,925)	6,635 (2,735)	33,330 (7,321)	10,999 (2,929)
88	GalNAcβ1,3GalNAcα-Sp8	24,610 (3,462)	17,169 (4,228)	11,034 (1204)	50,791 (5,749)	10,691 (3,009)
25	(GlcNAcβ1,3(GlcNAcβ1,6)GlcNAcβ1,4)GlcNAc-Sp8	20,305 (2,023)	6,148 (1,367)	8,080 (1,518)	50,775 (6,375)	2,992 (992)
21	GlcNAcβ-Sp0	15,621 (2,697)	14,972 (2,705)	3,377 (643)	34,825 (6,475)	5,354 (1,572)
22	GlcNAcβ-Sp8	22,909 (3,087)	12,289 (1,168)	2,514 (365)	31,863 (4,615)	10,738 (2,670)
174	GlcNAcβ1,6(Galβ1,3)GalNAcα-Sp8	12,541 (1,115)	6,017 (587)	3,492 (658)	46,930 5,852	3,206(997)
170	GlcNAcβ1,4Galβ1,4GlcNAcβ-Sp8	12,499 (1,921)	13,001 (2,039)	1,056 (190)	39,794 (6,245)	7,176 (2,338)
175	GlcNAcβ1,6GalNAcα-Sp8	11,120 (1,871)	6,683 (947)	2,432 (406)	43,848 (4,140)	26,522 (11,365)
166	GlcNAcβ1,3Galβ1,4GlcNAcβ1,3Galβ1,4GlcNAcβ-Sp0	10,917 (811)	7,563 (2,744)	3,599 (772)	43,425 (4,065)	4,334 (856)
121	Galβ1,3(GlcNAcβ1,6)GalNAcα-Sp8	10,256 (1,410)	6,215 (1,046)	3,850 (364)	50,893 (5,540)	3,195 (811)
176	GlcNAcβ1,6Galβ1,4GlcNAcβ-Sp8	9,029 (1,096)	5,372 (746)	5,119 (1,633)	40,973 (6,555)	2,269 (561)
163	GlcNAcβ1,3Galβ1,3GalNAcα-Sp8	7,713 (866)	10,655 (880)	3,999 (1,481)	47,984 (3,178)	31,661 (2,807)
169	GlcNAcβ1,4(GlcNAcβ1,6)GalNAcα-Sp8	4,520 (2,808)	12,960 (1,692)	1,680 (92)	38,200 (2,394)	19,830 (4,279)
158	GlcNAcβ1,2Galβ1,3GalNAcα-Sp8	5,082 (4,924)	1,016 (239)	75 (35)	305 (148)	563 (125)
23	GlcNH_2_β-Sp8	554 (117)	7,281 (1,506)	1,950 (1,358)	548 (256)	1,385 (464)
152	Galβ1,4GlcNAcβ-Sp0	1,703 (1,070)	1,974 (289)	249 (94)	19,659 (4,594)	1,279 (106)
173	GlcNAcβ1,4GlcNAcβ1,4GlcNAcβ-Sp8	848 (88)	2,860 (706)	1,690 (251)	5,689 (1,085)	2,418 (613)
148	Galβ1,4GlcNAcβ1,3Galβ1,4Glcβ-Sp0	391 (24)	1,090 (262)	919 (375)	3,942 (857)	663 (146)
213	Neu5Acα2,3(Neu5Acα2,6)GalNAcα-Sp8	4,527 (4,043)	4,331 (1,760)	1,388 (1,162)	1,229 (1,162)	1,717 (229)

Sp0 = -OCH_2_CH_2_NH-; Sp8 = -OCH_2_CH_2_CH_2_CH_2_NH-.

### 3.2. Validation of the Glycan Array Using Surface Plasmon Resonance

The best binding glycan structures, as well as non-binding ones, from the glycan arrays were subsequently evaluated for their affinity by SPR, using steady state equilibrium measurements identical to the ones performed to determine the millimolar-range affinities of F17G adhesins for β-d-GlcNAc (K_d_ = 1.2 mM, Table 2 and [18]).

**Table 2:** Steady-state affinities of variant F17G receptor-binding domains. The value of the dissociation constant K_d_ is expressed in mM for cases where a good fit could be obtained for at least three related experiments. * pNP-GlcNAc: *para*nitrophenyl β-GlcNAc, GlcNH_2_-MurNAc: glucosamine β1,4 *N*-actetylmuramic acid (from peptidoglycan), GM1 ganglioside: from ovine brain (Avanti Polar Lipids). Affinity ranges from high to low are indicated: +++ K_d_ = 10^−4^ M; ++ Kd = 10^−3^ M; + Kd = 10^−2^ M; - does not bind.

Carbohydrate	F17aG	F17bG	F17fG	F17eG
GlcNAcβ1,3Gal	+++ 0.66	++	+++	+++ 0.28
GlcNAcβ1,4GlcNAc (chitobiose)	++ 3.3	++	++	
GlcNAc ( *N*-acetylglucosamine)	++ 1.2	++	++	
Me β *N*-acetylglucosamine	++ 1.2			
Me α *N*-acetylglucosamine	+ 9.8			
GlcNAcβ1,2Gal	++ 4.2			++ 2.0
GlcNAcβ1,4Gal	++ 3.3			++ 2.7
GlcNAcβ1,6Gal	++ 2.8			++ 3.8
GlcNAcβ1,2Manα1,6(GlcNAcβ1,2Manα1,3)Man	++ 3.0			++ 3.0
Gal β1,4 GlcNAc	++			++
Galβ1,6GlcNAc	+	+	+	++
Galβ1,3GlcNAcβ 1,3Galβ1,4Glc	-			
Galβ1,4GlcNAcβ 1,3Gal β1,4Glc	+			
Fucα1,2Galβ1,3GlcNAcβ1,3Galβ1,4Glc	-			
Neu5Acα2,6Galβ1,4Glc	++			
pNP-GlcNAc *	+++	+	++	
*N*-acetylmannosamine	++	+	++	
*N*-acetylneuraminic acid	-	-	-	
Galβ1,3GalNAc	+			+
GlcNAc6SO_3_^−^	+		-	++
GlcNAc3SO_3_^−^	++	++	-	
GlcNAc6PO_3_^2−^	-	+	-	
GlcNH_2_-MurNAc *	+	+	-	
Neu5Acα2,3Galβ1,3(Neu5Acα2,6)GlcNAcβ1,3Gal β1,4Glc	+	+	-	
Le^Y^	-	-	++	
GM1 ganglioside *	-	+	-	
Neu5Acα2,3Galβ1,4GlcNAcβ1,2 Man	+			+
GalNAcβ1,4GlcNAcβ1-methoxy phenyl	++			

The results show that an *N*-acetyl group on the terminal saccharide is primordial for the interaction, whereas apparently several other saccharides (GalNAc, GlcNAc) can be supported. For those glycans with dissociation constants in the millimolar range or higher, there is very little significance in binding and these saccharides are unlikely to be part of a natural glycan receptor. For example, the Galβ1,4GlcNAc disaccharide displays very little affinity for F17aG, either using SPR or the CFG array (glycan number 152 in Table 1).

### 3.3. Co-Crystals of F17G Variants with Disaccharides

**Figure 3:**
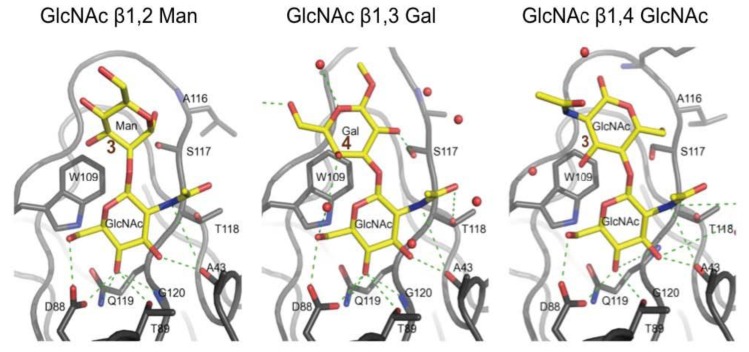
In F17bG crystal structures, the reducing end galactose (Gal) residue of GlcNAcβ1,3Gal (Φ = −67.3°/Ψ = 69.6°) stacks its hydrophobic B-face (at C4) onto the side chain of Trp109 (PDB entry 4K0O). The mannose (Man) residue in GlcNAcβ1,2Man occupies a similar position (Φ = −117.8°/Ψ = 104.4°), but stacks less efficiently because of its smaller apolar region (PDB entry 3FFO). The reducing GlcNAc residue in chitobiose (GlcNAcβ1,4GlcNAc) adopts a third, distinct orientation (Φ = −79.5°/Ψ = −142.6°) in the same site. The *N*-acetyl group of the second GlcNAc points away from the protein and is not likely to contribute to the binding (PDB entry 2BS7). Glycosidic angles Φ and Ψ are defined for 1,2 linkages: O5:C1-O:C2' and C1:O-C2':C3'; for 1,3 linkages: O5:C1-O:C3' and C1:O-C3':C4'; for 1,4 linkages: O5:C1-O:C4' and C1:O-C4':C5'.

A systematic soaking and co-crystallization study using synthetic disaccharides and substituted monosaccharides was undertaken, resulting in a number of crystal structures of F17G/ligand complexes [16]. In each of these complexes, a terminal, non-reducing GlcNAc occupies the primary binding pocket in the same orientation as observed in the initial F17aG structure with GlcNAc [18]. Functional groups conjugated to the β-anomeric oxygen of GlcNAc could provide additional stacking onto the hydrophobic region neighbouring the pocket (Figure 3). The binding mode of *para*nitrophenyl-subsituted β-d-GlcNAc to F17G (Protein Data Base (PDB) entry 3F64, [16]) and its affinity (Table 2) confirmed the contribution of hydrophobic packing interactions in this region.

The results from the glycan array profiling confirm GlcNAc as the non-reducing saccharide nesting into the binding site of F17G lectin domain (Figure 1, Figure 2). Consequently, the quest for the physiologically relevant receptor had its focus on oligosaccharides with a terminal GlcNAc residue. The reducing galactose residue of the disaccharide GlcNAcβ1,3Gal positions its hydrophobic B-face next to the Trp109 side chain (around C4, Figure 3). The reducing mannose residue of GlcNAcβ1,2Man occupies a similar position in the F17bG complex, but may stack less effectively because of the smaller apolar region (around C3, Figure 3) of mannose compared to galactose. Finally, the reducing GlcNAc residue in chitobiose (GlcNAcβ1,4GlcNAc, Figure 3) adopts a third, distinct orientation in the same general site in which the *N*-acetyl group of the second GlcNAc residue points away from the protein and is not likely to contribute to the binding interaction. This is congruent with similar affinity of F17G for chitobiose as for GlcNAc (Table 2).

**Figure 4:**
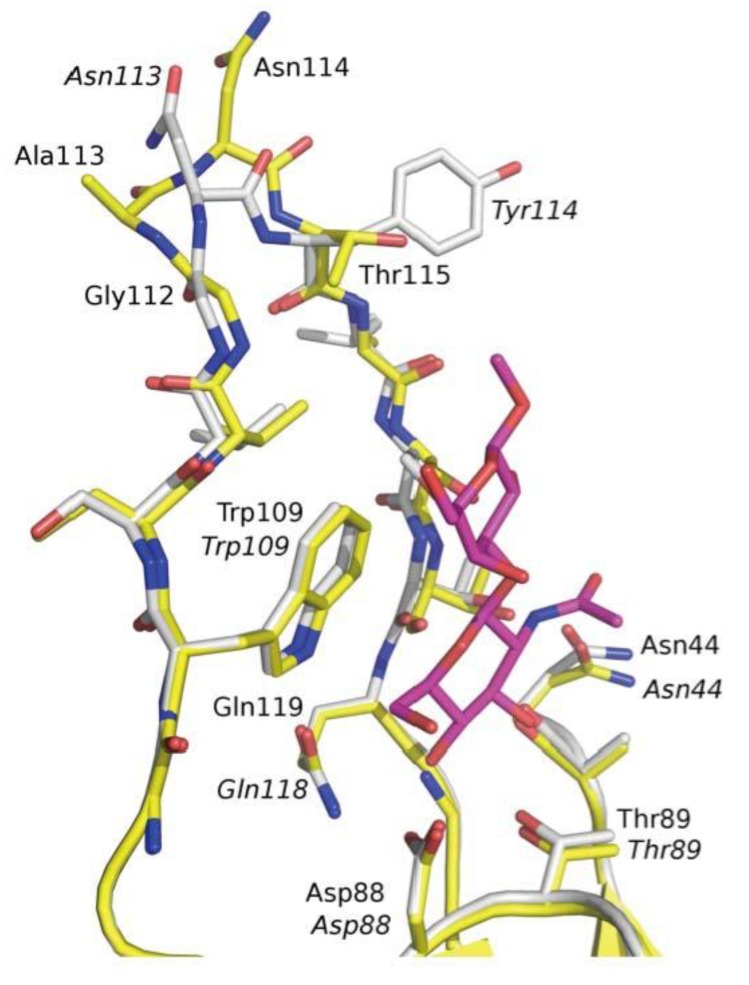
Superpositioning of the carbohydrate-binding sites of F17aG (yellow) and F17bG (white), highlighting the impact of the Ala113 insertion in F17aG. Residues of F17bG are labelled in *italics*. A bound GlcNAcβ1,3Gal ligand is shown in magenta to indicate the extent of the disaccharide-binding site.

In addition to these van der Waals contacts, there are many direct and water-mediated hydrogen bond interactions, although the number of additional bonds provided by the galactose of GlcNAcβ1,3Gal is limited to a strong hydrogen bond of the hydroxyl at position 2 to Ser117 (Figure 3). A water molecule (W109) between the GlcNAc and Gal residues of GlcNAcβ1,3Gal may influence the conformational ensemble of the ligand, by favoring the bound conformation. A superpositioning of the binding sites of F17aG and F17bG (Figure 4) based on the currently identified ligand does not explain the differences in tropism of the F17-fimbriated *E. coli* strains, a result that is thus congruent with the findings from the glycan arrays.

### 3.4. Shotgun Glycan Arrays for FedF and Whole F18-Fimbriated E. coli

**Figure 5:**
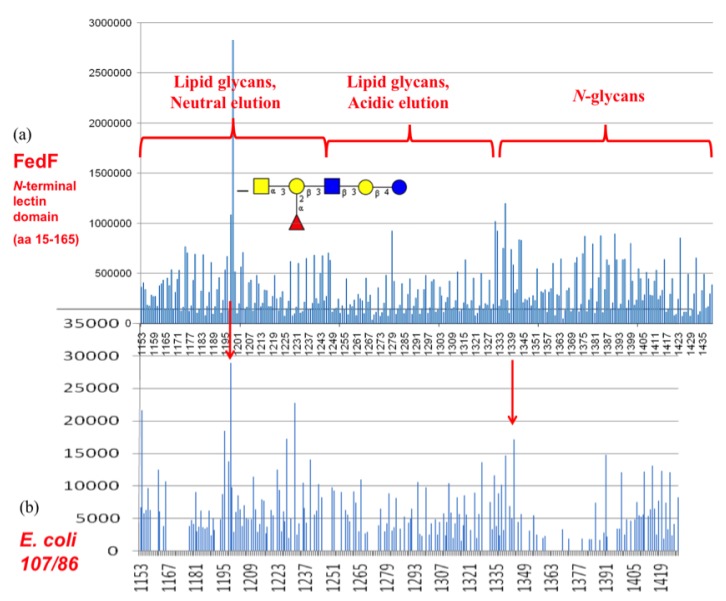
Shotgun glycan array screening (**a**) for the FedF lectin domain and (**b**) for F18 fimbriated *E. coli*.

The F18 fimbrial adhesin FedF had been tested on the CFG mammalian printed array version 2 (experiment 399) to hint at its potential binding specificity, which today has been very well characterized [25,38]. The data suggested very weak interaction of FedF with sulfated *N*-acetyl lactosamine structures and with sialylated blood group H type-2 trisaccharide [24]. It was also known that α1,2-fucosyltransferases acting on type-2 and/or type-1 precursor chains, to generate blood group determinants, were related to susceptibility to infections with F18-fimbriated *E. coli* [39]. Analysis of the CFG glycan array data did not reveal precise information on a fucosylated binding epitope and neither were there signals from exposure of FedF on the natural glycan array largely designed from *Schistosoma sp.* glycans [21]. Glycan arrays however only contain a subset of the eukaryotic glycome, therefore screening of the carbohydrate specificity of fimbrial lectins with a shotgun array, generated with glycans isolated from the targeted piglet intestinal cells as the starting material, appeared a logical choice to help identify the minimal glycan epitopes for those lectins. Based on our self-made shotgun glycan array, the functional blood group A hexasaccharide could be identified as a receptor for the purified FedF lectin domain (Figure 5). Despite increased background signals, a screening with F18-fimbriated bacteria again allowed to pinpoint the same functional receptor. The bound glycan structure was identified as GalNAcα1,3(Fucα1,2)Galβ1,3GlcNAcβ1,3Galβ1,4Glc by tandem mass spectrometry. Its identity was further confirmed by comparison of the tandem mass spectrometric fragmentation pattern with that obtained for a commercially obtained standard (details are given in Supporting Information, part 4).

### 3.5. F17G Variants Bind Acids and Sulfates on the Glycan Array Structures and in the Crystal

**Figure 6:**
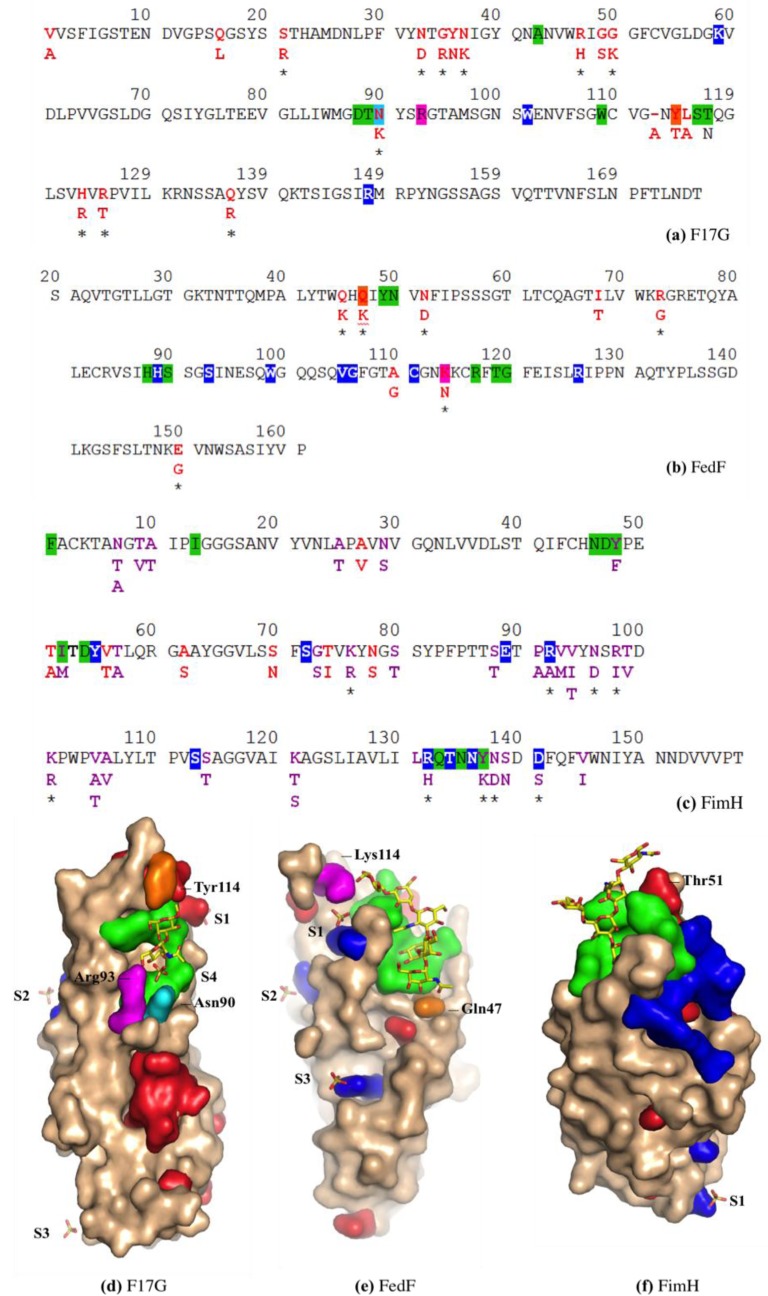
Amino acid analyses of *E. coli* strain-variable residues for the sequences (**a**, **b**, **c**) corresponding to the crystallized lectin domains (**d**, **e**, **f**) of F17bG (PDB 4K0O), FedF (PDB 4TST) and FimH (PDB 2VCO). Intraspecies variant residues are indicated in red, with those variable residues that also bind the glycan (near the non-reducing end) highlighted in orange. Residues ligating the recognized oligosaccharide are depicted in green. Sulfate-binding residues are highlighted in blue, in cyan when also variant or in fuchsia when also sugar-binding. Variations in charged residues are underpinned with a star (*). Sulfate ions S1–S4 have been identified in the different crystal structures.

Within the F17G adhesin family, variants a, b, c, d, e and f of *E. coli* share a minimum identity of 90% (159/177), with a maximum of 17 amino acid differences between F17G variants a and b (Figure 6a). The majority of the amino acid variations involve arginine or lysine, which consistently lead to either an introduction or a removal of a positive charge. The F17G lectin domain sequences have been aligned elsewhere [16], however residue 110 of F17bG has been confirmed in the crystal structure to be a cysteine, not serine, that forms a disulfide bridge with Cys53.

(**a**) Within the F17G adhesin family of *E. coli*, a maximum of 17 amino acid differences were found, between F17bG (the reference sequence) and F17aG adhesins, or a minimum identity of 90% (159/177). The majority of the differences are arginine or lysine mutations that consistently lead to either introduction or removal of positive charge. Sulfate-binding residues and the acetylated lysine 59 are indicated in blue. Asn90 is sulfate binding and variable and is colored red in a cyan box. (**d**) GlcNAcβ1,3Gal-binding residues are depicted in green, with Gal reaching out for variant residue Tyr114 (orange). Near the non-reducing end of the disaccharide, Arg93 (purple) is bridging the GlcNAc residue and the sulfate ion S4, whereas variable residue Asn90 (cyan) of F17bG, substituted for Lys90 in F17aG, is bridging the O4 hydroxyl of the reducing galactose and the sulfate ion S4. (**b**) The amino acid residues comprised in the FedF crystal structure are displayed with residue numbering of the mature protein. The reference amino acid sequence is for the *E. coli* F18 fimbrial adhesin AC that retains a minimum of 94% identity within the *Escherichia coli* FedF family. Again, variation in charged residues is indicated with a star (*). (**e**) FedF amino acids binding to GalNAcα1,3(Fucα1,2)Galβ1,3GlcNAcβ1,3Galβ1,4Glc are depicted in green. Variable residue Gln47/Lys is located near the terminal GalNAc residue of the type-1 blood group A hexasaccharide (onto green binding surface). Lys114 (purple) can bind the reducing glucose residue at the sphingolipid glycoconjugate as well as the sulfate, S1, identified in the crystal structure on the other side of the lectin domain joining the binding site. (**c**) Intraspecies variations (red) in the FimH lectin domain (*E. coli* UTI89 FimH as reference sequence compared with other FimH sequences [40]) occur only to a very limited extent (minimal identity 152/158 (96%) and involve changes among small hydrophobic and small polar residues. On the other hand, interspecies variations (magenta) with amino acid sequences of closest in identity between *E. coli* FimH UTI89 and *K. pneumoniae* (identities 139/158 (88%) and *K. oxytoca* 137/158 (87%) FimH often involve charged residues (*). (**f**) Three of the interspecies variant residues were found to bind sulfate in the crystal structure (of the FimH lectin domain in complex with oligomannoside-3: Arg92, Arg132 and Asp141 (blue) [26]. Oligomannose-3 binding residues are colored in green.

On the CFG mammalian printed arrays, the alpha1-acid glycoprotein scores high in binding all F17G variants, nevertheless the highest binding is for F17aG to HOOC(CH3)CH3-3-*O*-GlcNAcβ1,4GlcNAcβ-Sp10 on array version 4.1. F17aG is indeed highly positively charged, with three more lysines and one more arginine than F17bG [16]. Likewise, double sulfated Galβ1,4GlcNAc, [3OSO3]Galβ1,4[6OSO3]GlcNAcβ-Sp8, scores the tenth best binder for F17aG (version 4.1, exp. 2053). The same sugar is the 28th best binder for variant b (version 2, exp. 400), although caution should be excercised when interpreting the ranking because of the different versions of the arrays. The mutation in F17bG of Asn90 to Lys90 in F17aG could be at the basis of this observation, because a sulfate ion, S4, binds Asn90 in F17bG (Figure 6d). Indeed, in the novel co-crystal structure of F17bG in complex with GlcNAcβ1,3Gal, four sulfate ions could be modeled in their electron density (PDB entry 4K0O and Table S1 for data and refinement parameters). Interestingly, the addition of 10 mM GlcNAc-6-sulfate to crystals of F17bG instantly dissolves them. The sulfate ion S4 is situated near the hydroxyl group at position 4 of galactose, where the disaccharide could be extended, towards the reducing end, into a larger oligosaccharide sequence. F17bG has also been reported to bind disialylated lactose [41], a negatively charged glycan, however the fluorescence responses from sialylated candidates on the CFG array (glycan 213, Table 1) have very high standard deviations, leaving the nature and relevance of negatively charged modifications on F17G-binding glycans largely undefined. Variable residue Tyr114/Thr is located near the reducing end of the glycan and faces sulfate ion S1. The S2 sulfate ion binding site coincides exactly with a second GlcNAc binding site identified before in the crystal structure of F17cG, also called GafD [17].

### 3.6. FedF Binding to Blood Group Type 1 Core Glycans with Attraction to Acid Glycosphigolipids

The FedF receptor-binding domain has been found to bind to several different sulfated LacNAc and lactose glycans on a printed synthetic glycan array version 2 (264 glycans) by the CFG [20,24]. The most highly, triple, sulfated disaccharide (**1**) is here the best binder: Galβ1,4GlcNAc sulfated at positions 3', 6' and 6 (Table 3). Sulfation of the O6 hydroxyl (**2**) of GlcNAc is important, as well as *N*-acetylation on its position 2 (**3**) because the lactose form has lower affinity. Monosulfation on the non-reducing end galactose (one ion at either 3', 6' or 4') and lack of sulfation at the reducing end GlcNAc each decrease the fluorescence signal dramatically.

**Table 3:** CFG glycan array with the responses of the FedF lectin domain towards charged sugars. Sulfated sugars that gave RFU signals higher than their standard deviation on the CFG glycan array (experiment 399) are listed (RFU is the averaged Relative Fluorescence Units for the six replicates of each glycan and SEM (standard error of the mean) is the standard deviation (Stdev) divided by the square root of the six replicates).

candidate sulphated lactose structures		RFU	Stdev	SEM
**1**	[3OSO3][6OSO3]Galβ1,4[6OSO3]GlcNAc	10,388	3,852	1,573
**2**	[3OSO3]Galβ1,4[6OSO3]GlcNAc	6,806	1,854	757
**3**	[6OSO3]Galβ1,4[6OSO3]Glc	5,405	2,430	992
**4**	[3OSO3][6OSO3]Galβ1,4GlcNAc	4,733	1,157	472
**5**	[4OSO3][6OSO3]Galβ1,4GlcNAc	4,568	2,057	840
**6**	[3OSO3]Galβ1,4[6OSO3]Glc	4,073	1,515	618

In FedF from different *E. coli* strains, 6 in 8 mutations involve a change in charge (Figure 6b). In the crystal structure of FedF binding site complexed with Type 1 blood group oligosaccharide, Arg118 forms a hydrogen bond to the glycosidic linkage between the reducing glucose and the first galactose of the complexed ligand [25]. Residue 52, both as asparagine and aspartate, can form a salt bridge with Arg118 at an ideal distance of 4 Å. Another observed mutation is Gln47 to lysine. Gln47 is the closest residue to the hydroxyl at the O3 position of the non-reducing end galactose (for blood group B) or *N*-acetyl α-d-galactosamine (for blood group A) (orange in Figure 6b,e). Also Gln45 can be mutated to lysine, a residue that could possibly be interacting with a sialic acid α2,3 substituted at the non-reducing end of the oligosaccharide. Lys114 (purple) is a variant residue, recognizing the reducing glucose of the α1,2 fucosylated hexasaccharide (Figure 5a), that might bridge the sphingolipid, crossing over the edge of the FedF lectin domain, to the other side with sulfate binding site S1 (Figure 6e).

### 3.7. FimH Has a Charged Cradle over the Edge on the other Side of Its Oligomannoside Binding Site

*E. coli* intraspecies amino acid mutations in FimH comprise almost exclusively changes among small hydrophobic and small polar residues: only at the level of interspecies variation with *Klebsiella* species, charge variation comes into play (Figure 6c).The crystal structure of FimH bound to oligomannose-3 shows the accommodation of the non-reducing α1,3-linked mannoside in a deep binding pocket on the FimH surface (PDB entry 2VCO, Figure 6f). The minimal binding epitope is extended by interactions of the central mannose and the GlcNAc residues with the FimH tyrosine gate which protrudes from the binding pocket, resulting in a 100-fold increased binding compared to α-D-mannose [26]. Sulfate-binding residues in this crystal structure indicate a positively charged cradle on the other side of the tip of the adhesin (Figure 6f). An intraspecies variable residue, Thr51 (red), connects the reducing GlcNAc residue with this cradle. 

## 4. Discussion

Adhesins are critical determinants of the host range and tissue specificity of pathogenic bacteria. The adhesion of F17-fimbriated bacteria to the digiform intestinal microvilli depends on the F17G adhesin, which is located at the tip of the fimbria. The periplasmic chaperone F17D and the outer membrane usher F17C allow fimbrial assembly through donor strand complementation and exchange via the chaperone-usher pathway [4]. The F17C usher resembles most closely the HifD usher from *Haemophilus influenzae* Hif fimbriae, implicated in *otitis media* or middle ear infection [42], and recently classified under the γ4 fimbrial clade [43]. The closest relatives of the F17G adhesin are the fimbrial HifE adhesins from *Haemophilus aegyptius* (ATCC 11116) and *H. influenzae* (F3047). The glycan receptor for the HifE adhesin has been characterized as a long α2,6-sialylated glycosphingolipid [44].

The F17G adhesin was found to selectively bind glycan species with a terminal GlcNAc, typifying intestinal mucins. Our findings on both glycan arrays and the SPR measurements suggested oligosaccharide sequences carrying a terminal GlcNAcβ1,3Gal disaccharide with an affinity ranking for F17G of GlcNAcβ1,3Gal > GlcNAcβ1,6Gal > GlcNAcβ1,4Gal > GlcNAcβ1,2Gal (Table 1, Table 2). The affinity ranking is in agreement with earlier studies on bovine glycophorin and intestinal mucins of newborn calf, where GlcNAcβ1,3Gal was first identified as an F17G receptor. It was then suggested that GlcNAcβ1,3Gal is possibly also recognized as an internal sequence of larger oligosaccharides [45]. However, today it is known that terminal GlcNAc residues are present in large numbers on intestinal mucins [46]. Chitobiose, another inhibitor of F17G-mediated bacterial adhesion, is clearly lagging behind in affinity (Table 2). F17G can thus be ranked under those glycan-binding proteins that display high selectivity for GlcNAcβ1,3Gal, a common part of type 1 and type 2 blood group glycans. 

F17-fimbriated *E. coli* predominantly colonize neonatal animals, but are also a major causal agent (55%) of mastitis in bovines. The F17G ligand GlcNAcβ1,3Gal occurs universally; however, mostly internally in the sequence of poly-lactosaminyl glycans and blood group antigens. The *N*-acetyl glucosamine residue of GlcNAcβ1,3Gal may be non-substituted at the early life stage of calves, which are at the same time protected from bacterial infections by glycans secreted in the cow’s milk. Mucosal glycans, terminating on GlcNAcβ1,3Gal and newly identified as F17G receptors, are functional acceptors for mammalian glysosyltransferases [47]. This could explain the limited time window of infectivity of F17 *E. coli* strains in bovine calves and is indicative of a regulation by the degree of occurrence of the terminal GlcNAcβ1,3Gal receptors for pathogen association to cells [48]. Indeed, we show the binding of F17-fimbriated *E. coli* to be density-dependent (Figure 2b). One may therefore speculate that a high density of glycan receptors is required for adhesion of *E. coli* by the constitutively expressed F17 fimbriae to GlcNAcβ1,3Gal-terminating glycans, or that this motif is only part of the epitope of the recognized oligosaccharide sequence on epithelia.

Fimbrial adhesins are dependent on their glycan epitope for recognition and these glycan epitopes are age-, organ- and species-specific. Integration of our data from glycan array screening, from interaction studies using the purified adhesins, and from analysis of the crystal structure of the complexes of those fimbrial adhesins with glycan receptors in crystal structures gave new insights into the origin and the driving forces of bacterial fimbrial adhesion. In the FimH and FedF structures, a highly charged region is found that is in complex with a sulfate (S1), beyond the binding site of the reducing end sugar. Also in the newly defined F17bG crystals, there is not only a sulfate ion (S4) found close to the reducing galactose of GlcNAcβ1-3Gal, but also one (S1) over the edge of the lectin domain, much beyond the reducing galactose. This scenario is thus similar to what is found for FimH and FedF.

Charged amino acid variations in the vicinity of the reducing end of the receptor-binding pocket may be indicative of regio-selective binding in the colon. Indeed, the glycosylation of intestinal mucins is regio-specific and displays an acidic gradient through gradual increase in 3-*O*-sulfated galactose and sialic acid content along the intestinal tract [49]. Changes in sialylation, sulfation and fucosylation of mucosal glycans probably help to regulate the intestinal metabolism according to the processing site in the intestine, the kind of food, the age of the organism, *etc*. Sulfated sugars are therefore not an unusual but a normal component of the linings of the intestinal tract [50]. Negatively charged (glycan) residues play a role in water and electrolyte transport in the distal part of the colon, but the glycan arrays demonstrate that they are good targets for fimbrial adhesins, especially for those from enterotoxigenic *E. coli*, optimized to colonize the intestinal tract. The high mutation rate involving arginines and lysines (10 in 17 of the F17G and 6 in 8 of the FedF lectin domains, respectively) may thus present a functional adaptation among ETEC strains for the capture of glycan receptors that are increasingly modified with negative charges downstream the intestinal tract [49]. Strikingly, the fimbrial adhesin FimH does not undergo such charge changes. FimH recognizes high-mannosylated glycoproteins in the bladder, though it has not been characterized as a fimbrial adhesin from ETEC, but rather from uropathogenic opportuntists. In the bladder, the mucus layer is much thinner compared to in the intestinal tract. Interesting in this regard is that the *E. coli* variant EHEC O157:H7, causing hemolytic uremic syndrome (HUS) and being a binder in the intestinal tract, carries a mutation in the binding site of Asn135 towards the positively charged lysine. This mutation completely abolishes the ability of FimH to bind to bladder cells [40]. 

In other words, glycosphingolipid-binding adhesins, like F17G and FedF, have a structural diversity in electrostatic interactions that is not located inside the receptor-binding pocket, but rather beyond the reducing end of the oligosaccharide. The function of a polybasic loop near the ceramide-linked glucose residue of a blood group hexasaccharide was indeed found essential for the adhesion of F18-fimbriated bacterial to piglet enterocytes [25]. Interestingly, FedF-binding glycosphingolipids in intestinal mucosa of newborn pigs are the same as in adult pigs; however, the amount of acid glycosphingolipids in intestinal mucosa of newborn pigs is about seven times higher than the amount of the non-acid glycosphingolipids and about ten times higher than in adult pigs [51]. Neonatal piglets are thus extremely more receptive than older sucklings to F18-fimbriae-mediated infection and diarrhea. The maternal milk is likely to provide anti-bacterial glycans *in trans* as long as required, during the juvenile age of the pigs, but would be interrupted by weaning as early as 3 weeks after birth. Pathogenic *E. coli* strains could selectively profit from weaning by equipping the FedF lectin domain, in a postulated evolutionary process, with an increasingly higher number of positively charged residues. F18-fimbriated *E coli* may thus have developed into an extreme case of adaptation, to colonize weaned piglets presenting plentiful acidic glycosphingolipids and lacking the protection given by maternal milk oligosaccharides. 

The initial studies on F17G adhesins highlighted *N*-acetyl d-glucosamine as the only monosaccharide capable of inhibiting F17-mediated adhesion to intestinal microvilli [12]. High fluorescence signals on the CFG glycan array convincingly indicate that the GlcNAcβ1-3Gal disaccharide is part of the functional glycan receptor, for all the F17G lectin domain strain-dependent variants. This is despite the low millimolar affinity of F17G for GlcNAcβ1-3Gal-terminating oligosaccharides (K*_d_* = 660 μM), as confirmed in the SPR measurements, and a high concentration of the lectin domain (200 μg/mL) to be applied on the arrays. In contrast, no outstanding signal was observed for the 100 μg/mL deposits of FedF for neither of the two mammalian printed array versions 2 or 4.1 (experiments 399 and 2053, respectively). This is probably due to the absence of the fucosylated type-1 hexasaccharide on the CFG array and the inability, contrary to F17G (β-d-GlcNAc) and FimH (α-d-Man), of FedF to recognize simple terminal saccharides. Similarly, in the legume lectin family, most lectins can be classified on the basis of a significant affinity for a monosaccharide (Glc/Man, Gal, GalNAc, Fuc), whereas others display only complex oligosaccharide specificity [52]. 

While synthetic glycan arrays can offer pure and well-characterized glycan structures at well-determined concentrations, a shotgun approach could offer, as broadly as possible, the whole range of glycans from the relevant biological target. A shotgun glycan array may thus bring answers to the quest for sugar receptors recognized *in vivo* by lectins with a complex specificity of binding. The design of a shotgun glycan array from distal jejunum microvilli scrapings has been complementary to the CFG glycan array approach in the identification of the FedF receptor. This approach revealed the blood group type-1 sugars for FedF and for F18-fimbriated bacteria, findings (especially the latter one) of great diagnostic value for characterizing contamination with F18-fimbriated *E. coli* strains. Screening of this same array with F17G did not yield any information on larger ligands for the used lectin. Indeed, this array was not derived from newborn goat kids, calves or lambs, the typical target organisms for F17-fimbriated ETEC, but from a 3-week old piglet. 

In conclusion, we have identified intraspecies strain-dependent variation of fimbrial adhesins as a functional adaptation of enterotoxigenic *E. coli* to the acidic gradient on glycan receptors in the intestinal tract. ETEC adhesins tend to recognize longer glycan sequences, likely to improve their specificity and to bind selectively to a certain niche in the gut, where their chance for adhesion and survival is the highest. Charges may thus play a most important role in regio-selective bacterial adhesion. 

## Supplementary Files

Supplementary File 1 Supplementary (DOC, 881 KB)
