# Supplementary material for: Structural Sampling of Glycan Interaction Profiles Reveals Mucosal Receptors for Fimbrial Adhesins of Enterotoxigenic Escherichia coli
Source: Biology (Basel). 2013 Jul 1;2(3):894–917. doi: 10.3390/biology2030894 (PMC3960879; doi:10.3390/biology2030894)
Supplement: Supplementary File 1 — Supplementary (DOC, 881 KB) [file biology-02-00894-s001.doc]

*Biology* **2013**, *2*, 1-x manuscripts; doi:10.3390/biology20x000x

**OPEN ACCESS**

***biology***

**ISSN 2079-7737**

www.mdpi.com/journal/biology

Article

Structural Sampling of Glycan Interaction Profiles Reveals Mucosal Receptors for Fimbrial Adhesins of Enterotoxigenic *Escherichia coli*

Emanuela Lonardi ^1^, Kristof Moonens ^2,3^, Lieven Buts ^3,4^, Arjen R. de Boer ^1^,
Johan D. M. Olsson ^5^, Manfred S. Weiss ^6^, Emeline Fabre ^7^, Yann Guérardel ^7^,
André M. Deelder ^1^, Stefan Oscarson ^5^, Manfred Wuhrer ^1^ and Julie Bouckaert ^4,7^,*

^1^ Center for Proteomics and Metabolomics, Leiden University Medical Center, P.O. Box 9600, RC Leiden 2300, The Netherlands; E-Mails: e.lonardi@lumc.nl (E.L.); ardeboer@gmail.com (A.R.B.); a.m.deelder@lumc.nl (A.M.D.); m.wuhrer@lumc.nl (M.W.)

^2^ Structural & Molecular Microbiology, VIB Department of Structural Biology, Brussels 1050, Belgium; E-Mail: kristof.moonens@vub.ac.be

^3^ Molecular Recognition, VIB, Brussels 1050, Belgium; E-Mail: lievbuts@vub.ac.be

^4^ Vrije Universiteit Brussel, Pleinlaan 2, Brussels 1050, Belgium

^5^ Centre for Synthesis and Chemical Biology, University College Dublin, Belfield, Dublin 4, Ireland; E-Mails: johan.olsson@biochromix.com (J.D.M.O.); stefan.oscarson@ucd.ie (S.O.)

^6^ Helmholtz-Zentrum Berlin für Materialien und Energie, Institute for Soft Matter and Functional Materials, Macromolecular Crystallography (HZB-MX), Albert-Einstein-Strasse 15,
Berlin D-12489, Germany; E-Mail: manfred.weiss@helmholtz-berlin.de

^7^ Unité de Glycobiologie Structurale et Fonctionnelle (UGSF), Université Lille 1,
UMR8576 du CNRS, Villeneuve d’Ascq 59655, France;
E-Mails: emeline.fabre@univ-lille1.fr (E.F.); yann.guerardel@univ-lille1.fr (Y.G.)

***** Author to whom correspondence should be addressed; E-Mail: julie.bouckaert@univ-lille1.fr.

Received: 1 April 2013; in revised form: 15 May 2013 / Accepted: 17 May 2013 /
Published: 1 July 2013

1. CFG data and links

Printed array version 2 at the Consortium for Functional Glycomics [1] was used for glycan array screening of the variant F17G receptor binding domains. Slides were printed, with six replicates for each glycan, using glycan spotting concentrations of 100 μM, at the Consortium Carbohydrate Synthesis/Protein Expression Core D located at the Scripps Research Institute onto Schott Nexterion H slides (Schott Cat. No. 1070936B). Slide conditioning: preprinted slides were soaked in deionized water for 5 minutes at room temperature and dried under a stream of nitrogen. TSM buffer (20 mM Tris-HCl, pH 7.4, or MOPS 20 mM; 150 mM NaCl; 2 mM CaCl_2_, 2 mM MgCl_2_) was used as wash buffer (TSM buffer + 0.05% Tween 20) or binding buffer (TSM buffer + 0.05% Tween 20 + 1% BSA). (1) F17G was diluted to 200 μg/mL (Figure 1) in TSM binding buffer. (2) 50 μL of F17G was applied to the printed surface and coverslipped. (3) The slide was incubated, protected from light, in a humidified chamber for 1 hour at RT. (4) The slide was washed once in TSM wash buffer. 50 μL of the anti-F17G (10 μg/mL) polyclonal rabbit IgG were applied, coverslipped and incubated for 1 h at RT. (5) The slide was washed once in TSM wash buffer. Fifty μL of fluorescently labelled secondary goat anti-rabbit polyclonal antibodies (IgG-488, 5 µg/mL) were applied, coverslipped and incubated for 1 h at RT. (6) Slide was removed, followed by the coverslip, and washed for 10 s each in TSM wash buffer, TSM wash buffer lacking Tween 20, and deionized water. The slide was dried under a stream of nitrogen. (7) The binding image was read in a Perkin Elmer Microscanarray XL4000 scanner and tiff file of image stored. (8) Image analysis was performed using Imagene (V.6) image analysis software. (9) Raw data results files were generated in Excel format and uploaded to the Consortium database as .dat files. (10) Imagene data was uploaded to the database as .txt files.

**Figure 1.** Example of results from the glycan array, printed version 2, from the Consortium for Functional Glycomics, for F17eG at 200 μg/mL.


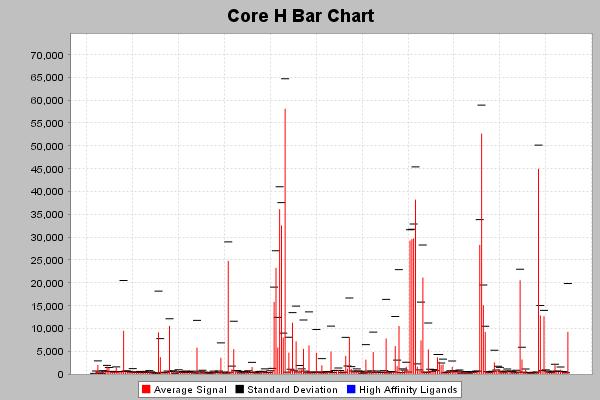


2. Crystal Data and links to the Protein Data Base

**Table 1.** Crystallographic data collections and refinements for the F17bG adhesin in complex with GlcNAcβ1-3Gal and the ligand-free FedF adhesion.

| **Protein** | **F17bG** | **FedF** |
| --- | --- | --- |
| **Ligand** | **GlcNAc(β1-3)Gal** | **Bromine/Sulfate** |
| **Beamline** | BW7B | EMBL/DESY X12 |
| **Space group** | P 6_2_ | P 2_1_ 2_1_ 2_1_ |
| **Unit cell** | *a* = *b* = 87.6, *c* = 57.0 | *a* = 36.2, *b* = 74.4, *c* = 98.9 |
| **Resolution range** | 43.8–2.15 (2.23–2.15) | 19.8–1.8 (1.85–1.80) |
| ***R_merge_*** | 0.069 (0.21) | 0.07 (0.2) |
| **Number of observed reflections** | 341,578 | 50,121 |
| **Unique relections** | 13,675 | 25,478 |
| **Completeness** | 99.9 (99.6) | 100.0 (99.9) |
| **<I/σ_I_>** | 39 (14) | 28.8 (12.9) |
| **Twinning fraction** | 0.40 | none |
| ***R*** | 16.80 | 16.45 |
| ***R_free_*** | 18.00 | 21.05 |
| **Geometry** |  |  |
| *Ramachandran plot (favoured/allowed regions)* | 93.6/98.8 | 97.96/100 |
| *Bond length r.m.s.d.* | 0.005 | 0.007 |
| *Angle r.m.s.d* | 0.889 | 1.149 |
| **Deposition code** | 4K0O | 4BWO |

3. Experimental Data and Scheme for the Synthesis of GlcNAc-containing Disaccharides used in the Investigation of the Specificity of F17G

**Scheme 1.** Synthesis of disaccharides GlcNAc(β1-2)Galβ1OMe (**10**), GlcNAc(β1-3)Galβ1OMe (**11**), GlcNAc(β1-4)Galβ1OMe (**12**) and GlcNAc(β1-6)Galβ1OMe (**13**).

Key: (*i*) NIS, AgOTf, CH_2_Cl_2_; (*ii*) (a) ethylenediamine, EtOH; (b) Ac_2_O, MeOH or pyridine (c) H_2_, Pd/C, MeOH/EtOAc/H_2_O.

**General methods:** Normal workup means drying the organic phase with MgSO_4_(s) or Na_2_SO_4_(s), filtering and evaporation of the solvent in vacuo at ~35 °C. CH_2_Cl_2_ was distilled over calcium hydride and collected onto predried 4A molecular sieves (MS). Thin Layer Chromatography (TLC) was carried out on 0.25 mm precoated silica-gel plates (Merck silica-gel 60 F_254_); detected with UV-abs (254 nm) and/or by charring with 8% sulfuric acid or AMC (ammonium molybdate (10 g) and cerium sulphate (2 g) dissolved in 10% H_2_SO_4_ (200 mL)) followed by heating to ~250 °C. FC means Flash Column chromatography using silica gel (Amicon, (0.040–0.063 mm)). ^1^H-NMR and ^13^C-NMR spectra were performed on a Varian 300 MHz or 400 MHz instrument at 25 °C, if no other temperature was given. Chemical shifts are given in ppm relative to solvent peaks (*δ* = 77.17 for ^13^C and *δ* = 7.26 for ^1^H) in CDCl_3_ or in D_2_O using acetone as an internal standard (*δ* = 30.9 for ^13^C and *δ* = 2.22 for ^1^H).

**Methyl 2-acetamido-2-deoxy-β-D-glucopyranosyl-(1,2)-β-D-galactopyranoside (10):** Ethyl 3,4,6-tri-*O*-benzyl-2-deoxy-2-phthalimido-1-thio-β-D-glucopyranoside [2] (**1**), (0.111 g, 0.18 mmol) and methyl 3-*O*-benzoyl-4,6-*O*-benzylidene-β-D-galactopyranoside [3] (**2**), (0.053 g, 0.14 mmol) were dissolved in dry CH_2_Cl_2_ (3 mL), 4 Å MS was added and the mixture was stirred under argon. After
20 min, the mixture was cooled to −20 °C followed by an addition of NIS (0.043 g, 0.19 mmol) and AgOTf (cat.). After 15 min, the mixture was neutralized with Et_3_N, diluted with CH_2_Cl_2_ (10 mL), filtered through Celite, washed with Na_2_SO_3_ (20% aq., 12 mL) and subjected to normal workup. FC (toluene/EtOAc 6:1) gave methyl 3,4,6-tri-*O*-benzyl-2-deoxy-2-phthalimido- β-D-glucopyranosyl-(1,2)-3-*O*-benzoyl-4,6-*O*-benzylidene-β-D-galactopyranoside (**6**, 0.087 g, 0.10 mmol, 74%); ^13^C-NMR (75.4 MHz, CDCl_3_): *δ* = 56.3 (2C), 66.0, 68.6, 69.0, 73.2, 73.8 (2C), 74.4, 74.4 (2C), 74.7, 74.9, 75.1, 79.2, 79.4, 98.0, 100.6, 102.3, 123.1, 126.2 (2C), 127.3, 127.6–128.5, 128.8, 129.5, 130.0 (2C), 133.2, 133.5, 137.7, 138.2, 138.2, 138.5, 165.9, 167.8 (2C). Compound (**6**) (0.083 g, 0.10 mmol) was dissolved in EtOH (2 mL) and ethylendiamine (320 µL, 4.8 mmol) was added. After stirring for 9 h at
80 °C, the solvent was removed under reduced pressure and the residue co-evaporated with toluene
(2 × 8 mL). A solution of the obtained amine in MeOH (2 mL) was cooled to 0 °C and Ac_2_O (1 mL) was added. After stirring 12 h, the mixture was concentrated and FC (CHCl_3_/MeOH 19:1) gave the 2-acetamido derivative (0.042 g, 0.055 mmol, 57%), which was dissolved in EtOH/EtOAc/H_2_O-mixture (3 mL) and Pd/C (10%) (cat.) was added. The mixture was stirred under hydrogen (100 psi) for 16 h, filtered through Celite and the solvents were evaporated. Purification on RP-FC (H_2_O) followed by
P2-BioGel size exclusion chromatography (1% BuOH in H_2_O) gave (**10**) (0.015 g, 0.039 mmol, 39% from (**6**)); ^13^C-NMR (100 MHz, CDCl_3_): δ = 22.4, 56.1, 57.1, 60.9, 61.1, 69.1, 70.0, 73.3, 73.9, 75.1, 76.1, 78.8, 101.8, 102.4, (-NHAc carbonyl peak not detected); ^1^H-NMR (400 MHz, CDCl_3_, 40 °C):
*δ* = 2.09 (s, 3H), 3.45–3.52 (m, 3H), 3.58–3.65 (m, 4H), 3.67–4.01 (m, 8H), 4.43 (d, 1H, *J* = 7.6 Hz), 4.85 (d, 1H, *J* = 8.4 Hz).

**Methyl 2-acetamido-2-deoxy-β-D-glucopyranosyl-(1,3)-β-D-galactopyranoside (11):** Donor
(**1**) [2] (0.084 g, 0.14 mmol) and methyl 2,4,6-tri-*O*-benzyl-β-D-galactopyranoside [4] (**3**), (0.048 g,
0.10 mmol) were coupled as described above for compounds (**1**) and (**2**) to give, after FC (toluene/EtOAc 19:1→9:1→6:1), methyl 3,4,6-tri-*O*-benzyl-2-deoxy-2-phthalimido-β-D-glucopyranosyl-(1,3)-2,4,6-tri-*O*-benzyl-β-D-galactopyranoside (**7**, 0.074 g, 0.072 mmol, 70%); ^13^C-NMR (75.4 MHz, CDCl_3_): *δ* = 56.7, 57.0, 69.0, 69.3, 73.5, 73.5 (2C), 73.7, 74.0, 74.7, 74.8, 75.0, 75.1, 76.2, 78.8, 79.7, 80.9, 99.4, 105.0, 123.2, 127.0, 127.2 (2C), 127.4–128.1, 128.4, 128.5 (2C), 128.5 (2C), 128.5 (2C), 133.6, 138.1, 138.2 (2C), 138.2, 138.9, 139.1, 168.0. Compound **7** (0.071 g, 0.069 mmol) was dissolved in EtOH (2 mL) and ethylendiamine (142 µL, 2.1 mmol) was added. After stirring 9 h at
70 °C the solvent was removed under reduced pressure and the residue co-evaporated with toluene
(2 × 8 mL). A solution of the amine in pyridine (1 mL) was cooled to 0 °C and Ac_2_O (1 mL) was added. After stirring for 2 h, the mixture was concentrated and FC (toluene/EtOAc 3:1 → 1:1) gave the 2-acetamido derivative (0.035 g, 0.037 mmol, 54%), which was dissolved in EtOH/EtOAc/H_2_O-mixture (3 mL) and Pd/C (10%) (cat.) was added. The mixture was stirred under H_2_ (100 psi) for 40 h, filtered through Celite and the solvents were evaporated. Purification on RP-FC (H_2_O), followed by P2-BioGel size exclusion chromatography (1% BuOH in H_2_O) gave (**11**) (0.011 g, 0.028 mmol, 40% from (**7**)).
^1^H-NMR spectra was in accordance with previously published data [5]; ^13^C-NMR (100 MHz, CDCl_3_): *δ* = 22.8, 56.4, 57.8, 61.2, 61.6, 69.0, 70.4 (2C), 74.3, 75.4, 76.4, 82.9, 103.4, 104.6, 175.6.

**Methyl 2-acetamido-2-deoxy-β-D-glucopyranosyl-(1,4)-β-D-galactopyranoside (12):** Donor
**1** [2] (0.128 g, 0.21 mmol) and methyl 2,3,6-tri-*O*-benzoyl-β-D-galactopyranoside [6] (**4**), (0.064 g, 0.13 mmol) were coupled as described above for compounds (**1**) and (**2**) to give, after FC (toluene/EtOAc 19:1 → 9:1), methyl 3,4,6-tri-*O*-benzyl-2-deoxy-2-phthalimido-β-D-glucopyranosyl-(1,4)-2,3,6-tri-*O*-benzoyl-β-D-galactopyranoside (**8**), (0.072 g, 0.067 mmol, 54%); ^13^C-NMR (75.4 MHz, CDCl_3_): *δ* = 55.2, 56.3, 64.4, 68.4, 68.9, 72.1, 72.7, 73.5, 74.8, 74.8, 74.9 (2C), 79.1, 79.5, 97.8, 101.3, 125.4, 127.4, 127.6–128.5, 129.0, 129.2, 129.6 (2C), 129.7 (2C), 129.9 (2C), 130.1 (2C), 130.3, 133.0, 133.1, 133.4, 138.2, 138.3 (2C), 164.6, 166.4, 166.7, 167.5, 167.9; Compound (**8**) (0.072 g, 0.067 mmol) was dissolved in EtOH (2 mL) and ethylendiamine (225 µL, 3.4 mmol) was added. After stirring 16 h at 70 °C the solvent was removed under reduced pressure and the residue co-evaporated with toluene (2 × 8 mL). A solution of the amine in MeOH (2 mL) was cooled to 0 °C and Ac_2_O
(1 mL) was added. After stirring 10 h, the mixture was concentrated and FC (CHCl_3_/MeOH 19:1) gave the 2-acetamido derivative (0.020 g, 0.030 mmol, 44%). This triol was dissolved in EtOH/EtOAc/H_2_O-mixture (3 mL) and Pd/C (10%) (cat.) was added. The mixture was stirred under H_2_ (100 psi) for 14 h, filtered through Celite and concentrated. Purification on RP-FC (H_2_O) followed by P2-BioGel size exclusion chromatography (1% BuOH in H_2_O) afforded (**12**) (0.011 g, 0.028 mmol, 41% from (**8**)); ^13^C-NMR (100 MHz, CDCl_3_): *δ* = 23.0, 56.4, 57.7, 61.3, 61.5, 70.7, 71.5, 73.4, 74.5, 74.9, 76.2, 76.6, 102.7, 104.4, 175.4; ^1^H-NMR (400 MHz, CDCl_3_): *δ* = 2.05 (s, 3H), 3.33–3.46 (m, 3H), 3.53–3.57 (m, 4H), 3.67–3.81 (m, 6H) 3.91 (d, 1H, *J* = 12.0 Hz), 4.07 (s, 1H), 4.30 (d, 1H, *J* = 7.6 Hz), 4.70 (d, 1H,
*J* = 8.4 Hz).

**Methyl 2-acetamido-2-deoxy-β-D-glucopyranosyl-(1,6)-β-D-galactopyranoside (13):** Donor
**1** [2] (0.068 g, 0.11 mmol) and methyl 2,3,4-tri-*O*-benzyl-β-D-galactopyranoside [5] (**5**), (0.040 g, 0.084 mmol) were coupled as described above for compounds (**1**) and (**2**) to yield, after FC (toluene/EtOAc 19:1 → 9:1), methyl 3,4,6-tri-*O*-benzyl-2-deoxy-2-phthalimido-β-D-glucopyranosyl-(1,6)-2,3,4-tri-*O*-benzyl-β-D-galactopyranoside (**9**), (0.044 g, 0.043 mmol, 51%). ^13^C-NMR (75.4 MHz, CDCl_3_): *δ* = 56.1, 56.7, 67.9, 68.7, 72.9, 73.4, 73.5, 73.7 (2C), 74.5, 75.0, 75.1, 75.1, 79.3, 79.5, 79.7, 82.2, 98.1, 104.8, 123.4, 127.5–128.6, 133.9, 138.1 (2C), 138.2, 138.6 (2C), 138.9 (no -NPhth signals detected); Compound **9** (0.044 g, 0.043 mmol) was dissolved in EtOH (2 mL) and ethylendiamine
(104 µL, 1.56 mmol) was added. After stirring 8 h at 70 °C the solvent was removed under reduced pressure and the residue co-evaporated with toluene (2 × 8 mL). A solution of the amine in MeOH
(2 mL) was cooled to 0 °C and Ac_2_O (1 mL) was added. After stirring 10 h, the mixture was concentrated and FC (toluene/EtOAc 3:1 → 1:1) gave the 2-acetamido derivative (0.023 g, 0.024 mmol, 56%), which was dissolved in EtOH/EtOAc/H_2_O-mixture (3 mL) and Pd/C (10%) (cat.) was added. The mixture was stirred under H_2_ (100 psi) for 14 h, filtered through Celite and concentrated. Purification on RP-FC (H_2_O) followed by P2-BioGel size exclusion chromatography (1% BuOH in H_2_O) gave (**13**) (0.0063 g, 0.016 mmol, 37% from (**9**)); ^13^C-NMR spectra was in accordance with previously published data [7]; H-NMR (400 MHz, CDCl_3_): *δ* = 2.03 (s, 3H), 3.44–3.57 (m, 7H), 3.63 (dd, 1H, *J* = 3.6, *J* = 10.0 Hz), 3.69–3.80 (m, 4H), 3.88–3.95 (m, 2H), 4.02 (m,1H), 4.29 (d, 1H, *J* = 8.0 Hz), 4.54 (d, 1H, *J* = 8.4 Hz).

4. Mass Spectrometric Identification of the Bloodgroup A Hexasaccharide Structure

The lipid glycan fraction giving the strongest signal on the FedF-probed microarray was subjected to reverse phase nanoLC-ion trap-MS/MS as described previously [8]. The major glycan identified in this fraction was an AA-labeled hexasaccharide of composition Hex3HexNAc2dHex1-AA. On the basis of its fragmentation, a blood group A hexasaccharide structure was assumed. The relevant blood group A hexasaccharide (type 1 chain) was ordered from Elicityl (www.elicityl-oligotech.com, order number Gly037-1; GalNAcα1,3(Fucα1,2)Galβ1,3GlcNAcβ1,3Galβ1,4Glc) and subjected to AA-labeling. In the following (Figure 2), the identity of the hexasaccharide present in the major FedF-bound shot gun array fraction was identified by comparison of its tandem mass spectra with those obtained for the blood group A standard.

**Figure 2.** Identification of a blood group A hexasaccharide as being the major component of the lipid glycan, neutral fraction predominantly recognized by FedF (see Figure 5 of the main manuscript for the shotgun glycan microarray data).

**Figure 2.** *Cont.*

**Figure 2.** *Cont.*

(**A**) Comparison of the ion trap MS/MS data obtained for the [M + H]^+^ species of the shotgun array fraction (upper panel) and the commercially obtained bloodgroup A hexasaccharide standard, both in AA-labeled form, observed at *m/z* 1178.7; (**B**) Comparison of the ion trap MS/MS spectra obtained for the corresponding [M + 2H]^2+^ species observed at *m/z* 589.9; (**C**) Comparison of the ion trap MS/MS spectra obtained for the corresponding [M+H+Na]^2+^ species observed at *m/z* 601.1. Blue diamonds indicate the position of the precursor in the tandem mass spectra. Bluesquare, *N*-acetylglucosamine; yellow circle, galactose; yellow square,
*N*-acetylgalactosamine; blue circle, glucose; red triangle, fucose; AA, 2-aminobenzoic acid; # doubly-charged species; * sodium adduct.

References

1. CFG Functional Glycomics Gateway, Glycan Array Data. Available online: http://www.functionalglycomics.org/glycomics/publicdata/primaryscreen.jsp/ (accessed on 10 September 2010).

2. Cirla, A.; McHale, A.R.; Mann, J. Synthesis of analogues of calicheamicin and neocarzinostatin chromophore. *Tetrahedron* **2004**, *60*, 4019–4029.

3. Wang, H.; She, J.; Zhang, L.H.; Ye, X.S. Silver(I) oxide mediated selective monoprotection of diols in pyranosides. *J. Org. Chem.* **2004**, *69*, 5774–5777.

4. Kohata, K.; Abbas, S.A.; Matta, K.L. Synthetic mucin fragments: Methyl 3-*O*-(2-acetamido-2-deoxy-β-D-galactopyranosyl-β-D-3-*O*-(2-acetamido-2-deoxy-3-*O*-β-D-galactopyranosyl-β-D-glucopyranosyl)-β-D-galactoyranoside. pyranosyl)-β-D-galactopyranoside. *Carbohydr. Res.* **1984**, *132*, 127–135.

5. Kajihara, Y.; Kodama, H.; Endo, T.; Hashimoto, H. Novel features of acceptor recognition by
ß-(1-4)-galactosyltransferase. *Carbohydr. Res.* **1998**, *306*, 361–378.

6. Garegg, P.J.; Oscarson, S. Synthesis of 6- and 6'-deoxy derivatives of methyl 4-*O*-a--galactopyranosyl-ß-galactopyranoside for studies of inhibition of pyelonephritogenic fimbriated *E. coli* adhesion to urinary epithelium-cell surfaces. *Carbohydr. Res.* **1985**, *137*, 270–275.

7. Abbas, S.A.; Kohata, K.; Matta, K.L. Synthetic mucin fragments. Methyl 6-*O*-(2-acetamido-2-deoxy-ß-glucopyranosyl)-ß-d-galactopyranoside, methyl 3,4-di-*O*-(2-acetamido-2-deoxy-ß-d-glucopyranosyl)-ß-d-galactopyranoside, and methyl *O*-(2-acetamido-2-deoxy-ß-d-glucopyranosyl)-(1-3)-*O*-ß-d-galactopyranosyl-(1-3)-*O*-(2-acetamido-2-deoxy-ß-d-glucopyranosyl)-(1–3)-ß-d-galactopyranoside. *Carbohydr. Res.* **1987**, *161*, 39–47.

8. Wuhrer, M.; Koeleman, C.A.; Hokke, C.H.; Deelder, A.M. Mass spectrometry of proton adducts of fucosylated *N*-glycans: Fucose transfer between antennae gives rise to misleading fragments. *Rapid Commun. Mass Spectrom.* **2006**, *20*, 1747–1754.

© 2013 by the authors; licensee MDPI, Basel, Switzerland. This article is an open access article distributed under the terms and conditions of the Creative Commons Attribution license (http://creativecommons.org/licenses/by/3.0/).
